# Understanding complex analytical data by a supervised correlation coefficient obtained from random forest

**DOI:** 10.1007/s00216-026-06629-5

**Published:** 2026-06-23

**Authors:** Stephan Seifert

**Affiliations:** Hamburg School of Food Science, Department of Chemistry, Universität Hamburg, Grindelallee 117, 20146 Hamburg, Germany

**Keywords:** Variable selection, Mass spectrometry, ICP-MS, Chemometrics, Statistics, Foods, Beverages, IR spectroscopy, Raman spectroscopy, Software

## Abstract

The analysis of complex analytical data, such as that obtained from mass spectrometry, nuclear magnetic resonance, or vibrational spectroscopic approaches, necessitates the employment of multivariate chemometric methods for the classification of biological samples or the quantification of individual components. In this context, accurate prediction of the samples is not the sole aim. The interpretation of the variables that contribute to this prediction is also of utmost importance. Consequently, a range of algorithms, e.g., based on partial least squares regression or random forest, have been developed for variable selection. One of these approaches, Surrogate Minimal Depth (SMD), utilizes surrogate variables, which are obtained in random forest, to incorporate variable relationships into the analysis of variable importance and to examine the mutual impact of variables on the model. The latter is expressed as a parameter that can be regarded as a supervised correlation coefficient. This paper aims to lucidly illustrate the selection of variables and the analysis of variable relationships with SMD. Furthermore, the method’s potential for analyzing complex analytical data will be highlighted by demonstrating its application to various types of analytical data.

**Graphical abstract:**

**Figure:**
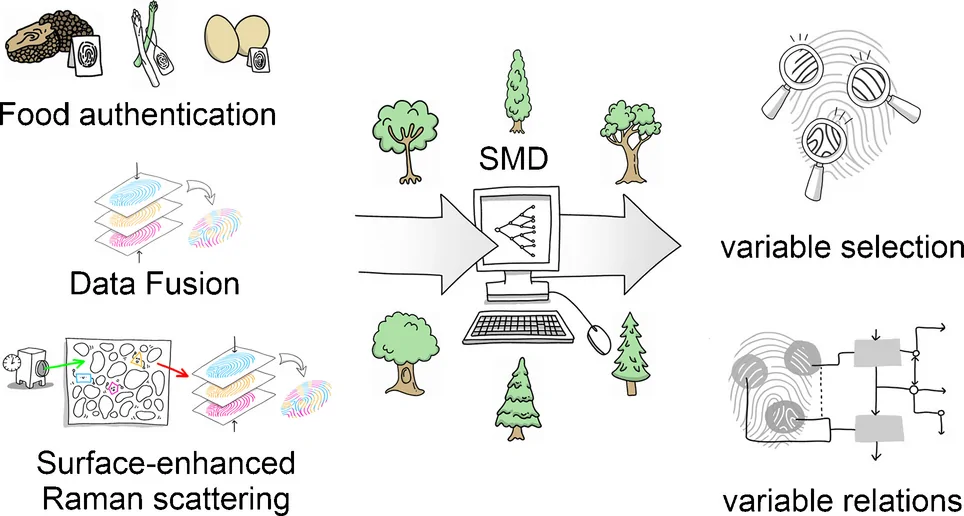

## Introduction

Complex biological materials, such as food, can be comprehensively characterized and classified using analytical techniques, which are, for example, based on mass spectrometry or various spectroscopic techniques. The data generated in this process is high-dimensional, meaning it is characterized by a large number of different variables from a relatively small number of samples. To exploit this data, multivariate methods are usually employed, meaning that all the variables in the dataset are analyzed simultaneously. These methods fall into two categories: unsupervised methods like principal component analysis, which are applied without additional information, and supervised methods, in which models are trained using samples with known assignments. In the latter case, it often makes sense to compare the performance of different algorithms, as their varying characteristics can affect the analysis differently depending on the specific features of the dataset being examined [1].

Supervised approaches based on latent variables, such as partial least squares regression (PLS), have the advantage of handling highly correlated data very well [2]. This is often advantageous when working with vibrational spectroscopic data. The non-parametric approach, random forest (RF), which is based on a large number of binary decision trees, also offers several distinct advantages, particularly when dealing with high-dimensional data [3]. RF has been demonstrated to be particularly effective in scenarios involving small sample sizes and a large number of uninformative variables [4]. Furthermore, RF provides internal validation. As each decision tree is built using a different subset of the training data generated via bootstrapping, the same dataset can be used for both training and validation simultaneously. For validation, only the “out-of-bag” samples, which were not used to train a given decision tree, are employed. Since random forests are much less tunable than other machine learning methods [5], meaning optimizing the hyperparameters has a relatively small impact on model performance, it is beneficial to omit this step. This has the huge advantage that the out-of-bag error from internal validation closely approximates the prediction error of an independent test dataset. Consequently, when analyzing a high-dimensional dataset, it is possible to use all samples for training while still obtaining a realistic estimate of the model’s performance on unseen data. Due to the advantages of RF, it is applied in many fields, including food authentication [6–8] and health data analytics [9, 10].

When applying supervised multivariate methods, it is not only important to obtain models with high predictive performance on unknown samples, but also to understand the underlying relationships that lead to these predictions. When PLS is used, the loadings can be used for interpretation. In addition, various methods have been developed for the selection of relevant variables [11]. Similarly, also, RF-based methods enable the differentiation between important and unimportant variables [12]. All of these methods determine the importance of each variable individually and do not take into account the relationships between them.

The methodological expansion of Surrogate Minimal Depth (SMD) is that, rather than considering the variables individually, it analyzes their mutual impact on the RF model [13]. This concerns the assessment of the importance of variables in the random forest model, in which variable relationships are taken into account. Furthermore, the agreement of the variables in the individual splits of the decision trees is evaluated across the RF to calculate a relation parameter that quantifies the mutual impact of the features. The analysis of this parameter goes beyond a simple analysis of the importance of individual variables and enables a comprehensive interpretation of the relationships underlying the model. This, in turn, provides an overall characterization of the variable and sample properties involved. This paper provides a comprehensive explanation of SMD, focusing on diagrams rather than formulas. It also highlights various applications in which this analysis has revealed interesting relationships.

## Surrogate Minimal Depth (SMD)

**Fig. 1:**
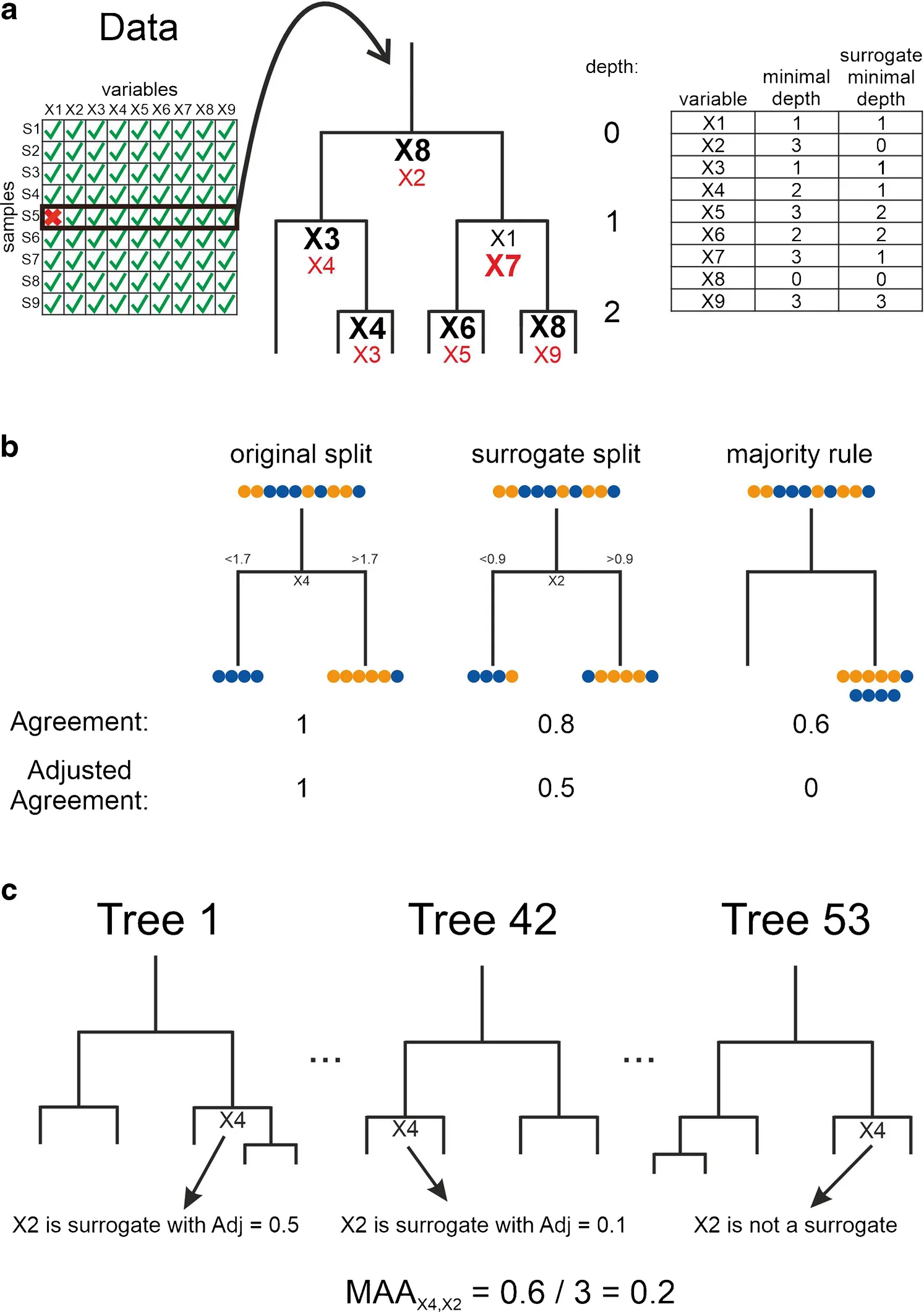
Graphical explanation of the basic functioning of surrogate variables and SMD. **a** shows that a decision tree can be used to predict a dataset (S5) with missing values when surrogate variables are employed (left). Furthermore, a single decision tree is used as an example to show how the importance measures “Minimal Depth” and “Surrogate Minimal Depth” are determined (right). **b** illustrates the parameters “agreement” and “adjusted agreement,” which are used to determine surrogate variables and splits. While the agreement compares the performance of potential surrogates with that of the original split, the adjusted agreement also makes a comparison with the majority rule, an alternative approach of dealing with missing values in decision trees. **c** For relation analysis in SMD, the adjusted agreement for the respective combinations of variables (in this case, variables X4 and X2) is averaged across the entire RF, resulting in the mean adjusted agreement (MAA), which can be seen as a supervised correlation coefficient

Surrogate variables were already included in Breiman’s original implementation of the random forest algorithm [3]. When training the decision trees that comprise a random forest, alternative splits were identified that could serve as optimal replacements for the original splits. This enables the model to be applied to datasets with missing variables (see Fig. 1a, left), which can be the case, for example, with medical patient data. In SMD, however, surrogate variables are used for a different purpose [13]. They are combined with the metric minimal depth, which determines the relevance of a variable based on the position at which it first appears in the decision trees (Fig. 1a, right). In SMD, the importance of a variable is thus determined by its initial appearance as a primary split or surrogate variable.

**Fig. 2:**
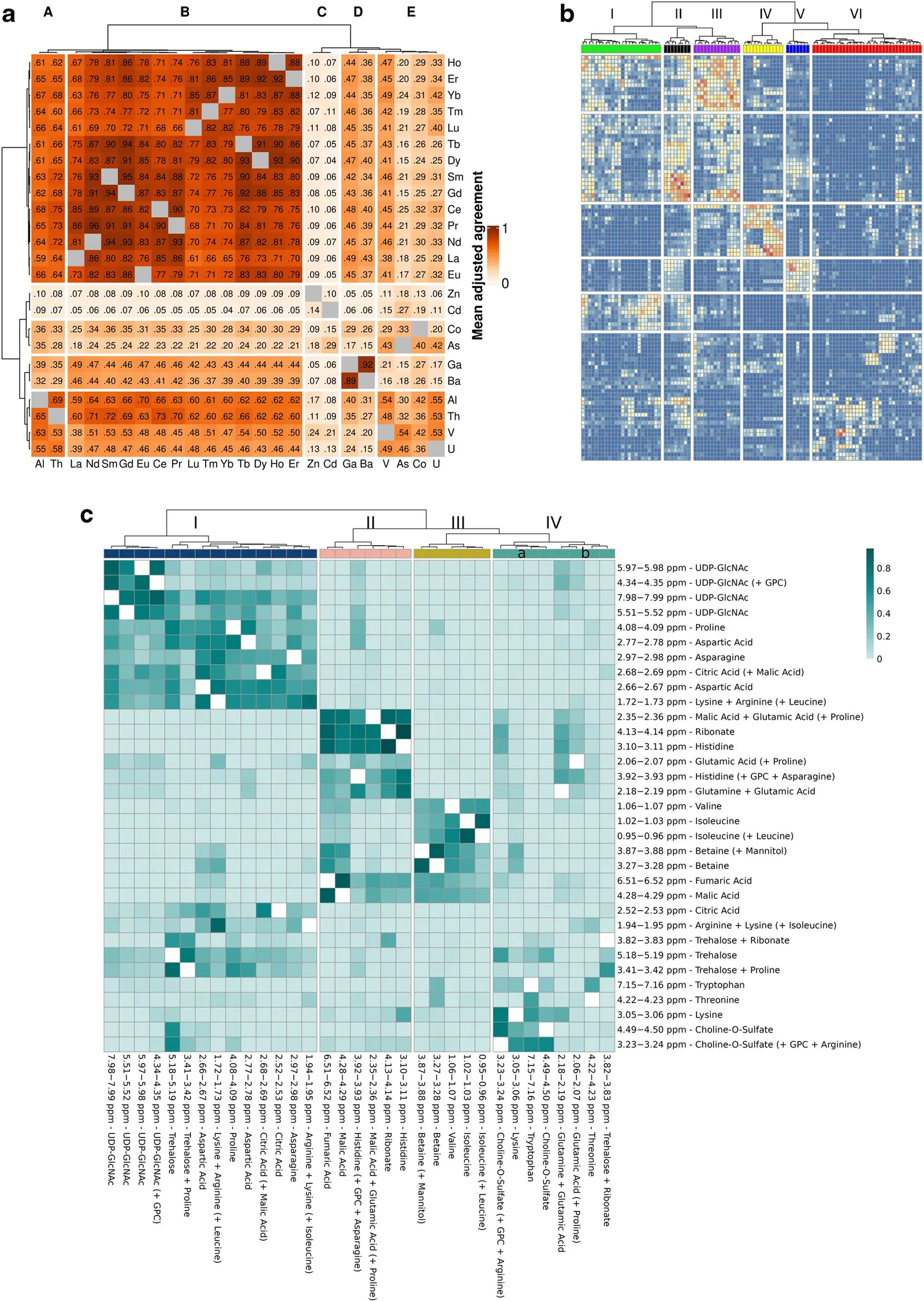
Application of the SMD relation analysis to ICP-MS [14] (**a**), LC-MS [15] (**b**), and NMR data [16] (**c**) to predict the geographical origin of asparagus (**a**, **b**) and the taxonomic variety of truffles (**c**). Boxplots of the variables for each of the clusters obtained using hierarchical clustering with Euclidean distances and Ward’s algorithm can be found in the supplementary files of the respective publications. Figures are reproduced under CC-BY license

It is evident that the capacity of a variable to substitute for another in the decision trees of a random forest offers insight into the nature of their relationship. Figure 2b shows how surrogate variables and splits are determined when training a decision tree: The parameter “adjusted agreement” compares the distribution of samples of the original split with those of possible surrogate splits and the so-called majority rule, in which all samples are simply assigned to the direction taken by the majority of samples. The surrogate variables and splits with the highest value above 0 are used. In SMD, the pairwise variable relationship is quantified by using the parameter adjusted agreement, and the mean adjusted agreement (MAA) is determined over the entire random forest (Fig. 1c). Because this metric is based on the supervised random forest model, it reflects the mutual association of the variables on the outcome. For this reason, MAA can also be referred to as a supervised correlation coefficient, which facilitates to thoroughly evaluate the complex relationships between variables and outcomes.

Although this increases the computational requirements, it is generally recommended that a large number of deep trees be trained when performing SMD, to ensure that the analysis is based on a sufficient number of nodes. For the SMD-specific parameter s, i.e., the maximum number of surrogate variables identified for each split, a value of 5% of the total number of variables is recommended. However, for datasets with relatively few variables, this value should be increased.

## Application for food authentication

SMD was applied to various types of analytical data to analyze the mutual impact of variables for food authentication [14–19]. To clearly visualize the identified relationships, the observed values of the supervised correlation coefficient, which are determined for each pairwise combination of the selected variables, are usually grouped using cluster analysis and presented in a heatmap. The subsequent sections will provide a more detailed examination of the results of inductively coupled plasma-mass spectrometry (ICP-MS), liquid chromatography-mass spectrometry (LC-MS), and nuclear magnetic resonance (NMR) spectroscopy analyses (Fig. 2).

Five clusters of elements were obtained for ICP-MS analysis to determine the geographical origin of asparagus (Fig. 2a) [14]. These clusters enable the selected elements to be distinguished based on their influence on the model. This means that the elements within each cluster show a very similar distribution of geographic origins; gallium and barium in cluster D even provide nearly identical information for classification. The similar behavior of the elements can be attributed to various factors. For example, there may be chemical similarities, as is the case with the rare earth elements in cluster B. However, it is also possible that the relationships can be attributed to different environmental factors affecting the asparagus plants. Since both aluminum and thorium in cluster A are influenced by soil pH, the difference observed for samples from Spain and the Netherlands, and those from the other analyzed geographical origins, could be attributed to this environmental factor.

The analysis of the same asparagus samples using LC-MS resulted in the formation of six clusters, comprising metabolites with similar information for classification (Fig. 2b) [15]. In many cases, the relationships obtained can be interpreted in such a way that the data has been classified into biologically relevant groups. Cluster IV, for example, contains various (18:2)-phytosterol esters involved in the sterol biosynthesis pathway. Therefore, SMD analysis identified an entire pathway to be relevant to differentiate between samples from Germany and the Netherlands and those from other countries of origin. This knowledge can be applied to make the classification more robust and therefore less susceptible to falsification adaptations.

**Fig. 3:**
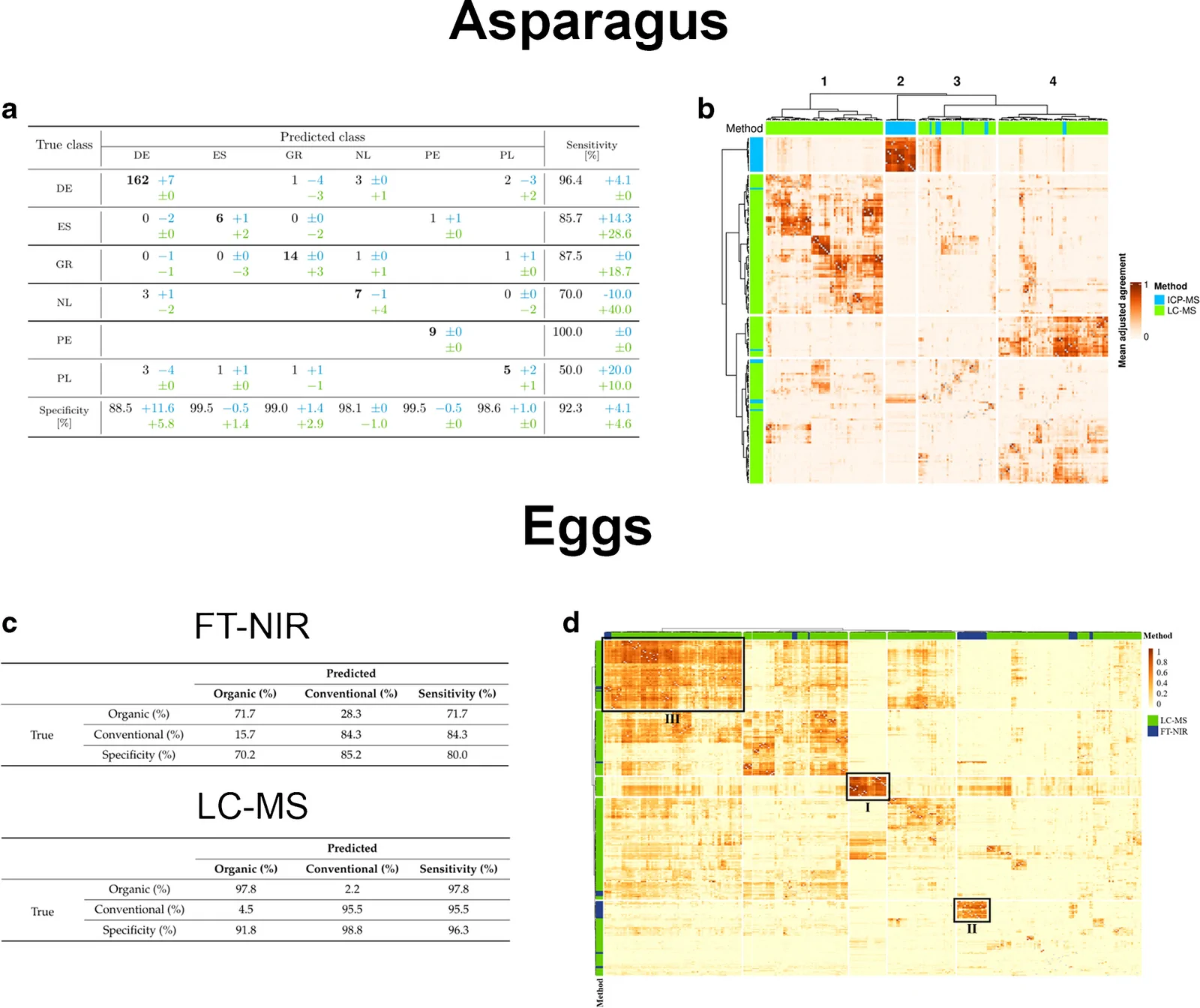
Application of SMD to the data fusion of ICP-MS and LC-MS data to predict the geographical origin of asparagus (**a**, **b**) [14] and the data fusion of NIR and LC-MS data to differentiate organically and conventionally produced eggs (**c**, **d**) [21]. The accuracy of the random forest models (**a**, **c**) and the heatmaps for the relational analysis (**b**, **d**) are shown. **a** shows the prediction results of the model based on the fused data compared to the models based on the individual ICP-MS data (blue) and the individual LC-MS data (green). Further information on the selected variables, which have been grouped here using hierarchical clustering with Euclidean distances and Ward’s algorithm, can be found in the supplementary files of the respective publications. Figures are reproduced under CC-BY license or redesigned (**a**)

The application of SMD to NMR data from truffles also resulted in the formation of biologically meaningful groups of variables with similar influence on the random forest model (Fig. 2c) [16]. Similar amino acids, such as isoleucine, leucine, and valine, are grouped together and provide similar information for classification. However, for the analysis of NMR data, another advantage of SMD relation analysis becomes evident. In NMR data processing, it is common practice to create buckets that group together adjacent variables. In this process, signals from different metabolites may end up in the same bucket, making it impossible to determine which signal and which metabolite the relevant information comes from. The bucket between 3.41 and 3.42 ppm, for example, was assigned to trehalose and proline (Fig. 3c). Since it is highly related to a feature between 5.18 and 5.19 ppm, which was exclusively assigned to trehalose, it can be concluded that most of the relevant information in the previous bucket must come from trehalose and not proline.

In summary, the supervised correlation coefficient of SMD makes it possible to identify relationships in complex analytical data that can be attributed to chemical similarities, similar biological causes (such as environmental influences or shared pathways), as well as to determine which components of overlapping signals are relevant.

## Data fusion

The application of SMD to analyze variable relations across different datasets has also been demonstrated to be very useful [20]. SMD can be used for different purposes in this context. One of them is to examine how variables interact to improve predictions through fusion, which was applied for the analysis of the ICP-MS and LC-MS datasets described above to distinguish the geographical origin of asparagus. Using the data in a low-level fusion resulted in slightly more accurate predictions than using the individual datasets (Fig. 3a). This was particularly evident in the case of the smaller groups, namely Greece (GR), the Netherlands (NL), and Spain (ES). The results of the SMD analysis demonstrated how the variables from the two datasets complement each other to achieve this improved classification, particularly for these groups (cluster 3 in Fig. 3b).

Another objective was pursued by applying SMD to a fused dataset of LC-MS and near-infrared (NIR) spectroscopy data for the differentiation of organic and conventionally produced eggs. The interpretation of NIR data is generally complicated by the presence of overlapping signals from various molecules, making it difficult to unambiguously assign specific molecules. The model performance based on NIR data was significantly worse than that based on LC-MS data (Fig. 3c), and SMD was applied to the fused data to analyze the cause of this poorer performance. It is evident that NIR variables are also present in the lipid cluster III, indicating that NIR can be utilized for the detection of differences in lipid composition (Fig. 3d). However, cluster I, which shows differences in carotenoid composition, consists exclusively of LC-MS variables. These were essential for accurate differentiation based on LC-MS data. Therefore, the difference in performance between these two methods is likely due to the fact that carotenoids cannot be detected using NIR.

## Surface-enhanced Raman scattering (SERS)

SERS refers to the phenomenon whereby Raman signals are amplified by many orders of magnitude in the immediate vicinity of plasmonic nanostructures, such as gold or silver nanoparticles. When this method is used to analyze complex biological samples like cells, the resulting spectra can be very different depending on the presence of the molecules in close proximity to the nanoparticles at the time of measurement [22]. Consequently, when a cell is repeatedly measured, as is customary, varying spectra are obtained that reflect the nanoparticles’ constantly changing environment. Although the data obtained offers enormous potential for the precise analysis of cellular processes, the vast amount of varied information it contains poses a major challenge for data analysis [23].

**Fig. 4:**
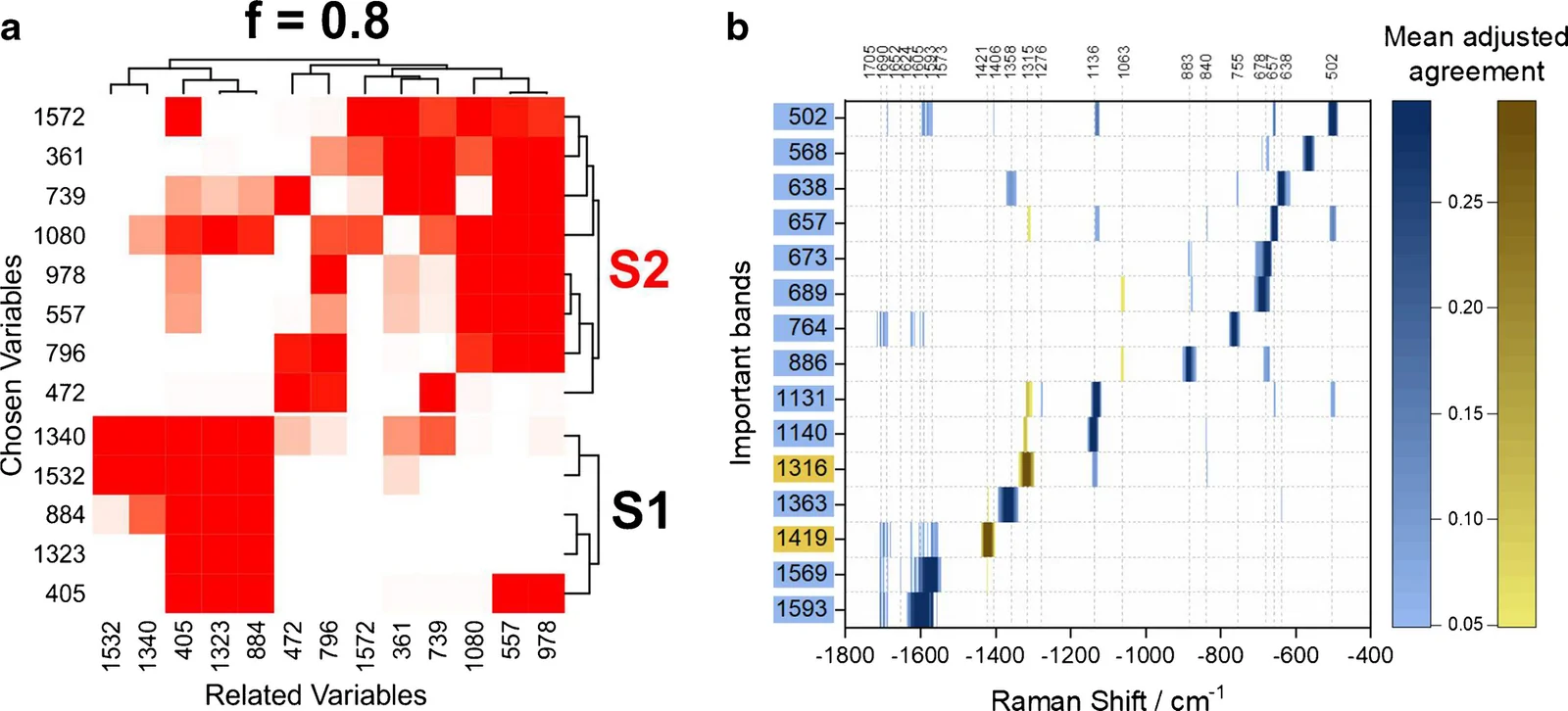
Application of the SMD relation analysis to SERS data. **a** shows the results of a simulation study demonstrating that co-occurring signals (S1 and S2) can be grouped by the supervised correlation coefficient [24]. In **b**, the application to SERS spectra from 3T3 cells incubated with ceramide is shown [28]. Based on the relationship between selected variables of important bands on the *x*-axis and variables assigned to protein and lipid bands (shown in blue and yellow, respectively), conformational changes in proteins were associated with membrane remodeling processes in endolysosomes. Figures are reproduced under CC-BY license

A simulation study demonstrated that random forest methods, and in particular the supervised correlation coefficient from SMD, can be effectively applied to the analysis of SERS data [24]. In this study, two datasets of SERS spectra were simulated. The majority of the signals were present in both datasets, but some of them were unique to each dataset, and these unique signals always occurred together (with varying overall frequency, expressed by the parameter f). SMD was able to correctly select the signals as relevant for discrimination of the two datasets and to group the simultaneously occurring signals (see Fig. 4a).

When applied to experimental SERS data in the context of cell analysis, co-occurring signals can be attributed to molecules that are present simultaneously within the focal volume of the measurement and are therefore likely to be interacting within the cell. Using SMD, we were able to investigate such interactions in a number of studies. These include analyzing the interactions between lipids and antidepressants that lead to lipid accumulation in cells [25] and investigating the metabolism of ceramides and the molecular consequences of cellular ceramide imbalance [26–28]. This approach was particularly helpful when analyzing lipid interactions, as these often exhibit very weak signals that prevent investigation using other methods.

As the results obtained from the SMD relation analysis are complex, it is challenging to present them clearly. Figure 4b illustrates how this can be achieved. First, the selected signals are aggregated so that one representative is used for each relevant band. For each of these, the pairwise supervised correlation coefficient is plotted, with both the relevant bands and the signals of the corresponding relationships colored according to the molecular group (in this case, proteins or lipids). Consequently, it becomes immediately evident which of the examined relationships reflect interactions between different molecular groups. This, in turn, made it possible in this study to link conformational changes in proteins to alterations in the endolysosomal membrane.

## Outlook

This paper provides a thorough examination of the analysis of variable relationships using the random forest-based approach SMD. Various application examples demonstrate the potential of the resulting supervised correlation coefficient to contribute to the identification of complex relationships in analytical data. In particular, combining this approach with SERS data acquired from living cells holds promise for illuminating various complex biological processes. A further development of the SMD methodology, which enables the analysis of relationships involving categorical variables, further expands the scope of its application, particularly in the medical field, as it enables, for example, the analysis of gene expression data or clinical variables [29, 30].

## Data Availability

No data requiring publication was generated as part of this overview. The R package, which includes both Surrogate Minimal Depth (SMD) for selecting relevant variables and analyzing variable relationships, as well as its further development into Mutual Impurity Reduction (MIR) and Mutual Forest Impact (MFI), can be found here: https://github.com/AGSeifert/RFSurrogates.
